# Antegrade insertion of a double J catheter in the treatment of malignant ureteral obstruction: a retrospective analysis of the results obtained with a modified technique at a university hospital

**DOI:** 10.1590/0100-3984.2019.0090

**Published:** 2020

**Authors:** Rômulo Florêncio Tristão Santos, Tiago Kojun Tibana, Edson Marchiori, Thiago Franchi Nunes

**Affiliations:** 1 Universidade Federal de Mato Grosso do Sul (UFMS), Campo Grande, MS, Brazil.; 2 Universidade Federal do Rio de Janeiro (UFRJ), Rio de Janeiro, RJ, Brazil.

**Keywords:** Urinary catheterization/instrumentation, Stents, Ureteral obstruction, Radiology, interventional, Urologic neoplasms, Inserção anterógrada, Cateter duplo J, Obstrução ureteral, Radiologia intervencionista, Neoplasias urológicas

## Abstract

**Objective:**

To analyze the results obtained with a modified antegrade double J catheter insertion (JJ stenting) technique in patients with urinary tract obstruction due to malignancy.

**Materials and Methods:**

This was a retrospective analysis of data collected from patients undergoing antegrade JJ stenting for malignant ureteral obstruction in the interventional radiology department of our institution between March 1, 2017 and May 31, 2019.

**Results:**

Antegrade JJ stenting was performed in 32 patients (20 women and 12 men). The mean age was 66.2 years among the females and 61.5 years among the males. A total of 53 antegrade JJ stenting procedures were performed. The procedure was successful in 50 cases and failed in 3 (due to migration of the double J catheter in 2 and due to technical failure in 1). Complications occurred in 3 patients (low back pain, in 1, subcapsular hematoma, in 1, and pyelonephritis, in 1). The procedure time ranged from 14 min to 55 min.

**Conclusion:**

In patients with ureteral obstruction due to malignancy, antegrade JJ stenting is safe and effective. The technique selected in our study is easily reproduced and can be performed by a trained professional.

## Abstract (pt)

**Objetivo:**

Análise retrospectiva dos resultados de uma técnica modificada de inserção anterógrada de cateter duplo J em pacientes com obstrução do trato urinário por causas oncológicas.

**Materiais e Métodos:**

Coleta e análise retrospectiva de dados de pacientes submetidos a inserção anterógrada de cateter duplo J por obstruções ureterais neoplásicas no departamento de radiologia intervencionista da instituição, entre 1º de março de 2017 e 31 de maio de 2019.

**Resultados:**

No total, 32 pacientes foram submetidos a inserção anterógrada de cateter duplo J, sendo 20 mulheres e 12 homens. A média de idade foi de 66,2 anos para o sexo feminino e 61,5 anos para o sexo masculino. Cinquenta e três inserções anterógradas de cateter duplo J foram realizadas. O número de procedimentos bem sucedidos foi 50 e houve 3 falhas (2 migrações do cateter duplo J e 1 insucesso técnico). As complicações ocorreram em 3 pacientes (1 dor lombar, 1 hematoma subcapsular e 1 pielonefrite). O tempo de procedimento variou entre 14 e 55 minutos.

**Conclusão:**

A inserção anterógrada de cateter duplo J em pacientes com obstrução ureteral por causas malignas é eficaz e segura. A técnica descrita no nosso estudo é de fácil reprodução, podendo ser executada por um profissional capacitado.

## INTRODUCTION

Ureteral obstruction is a heterogeneous clinical entity, and determining the ideal decompression method often represents a challenge for the primary care physician. Such obstructions can stem from an intrinsic primary neoplasm such as cancer of the bladder or prostate, as well as from the extrinsic secondary involvement of other malignancies, most commonly of colorectal or gynecological origin^(1-3)^. The therapeutic objectives of draining the upper urinary tract in cases of malignancy are symptomatic relief, maintenance of renal function, reducing the length of the hospital stay, and minimizing the negative impact on patient quality of life^(1-5)^.

There are no clear guidelines regarding the ideal methods for decompressing the urinary tract in the management of ureteral obstructions^(1)^. Double J catheters are normally inserted by a retrograde pathway under cystoscopic guidance. That approach, however, can be difficult, especially in patients with anatomical distortion of the bladder wall or malignant ureteral obstructions with involvement of a long ureteral segment, because of the technical inability to advance the guidewire beyond the point of obstruction; in such cases, the only options are percutaneous nephrostomy and antegrade insertion of a ureteral catheter^(6)^. The disadvantages of nephrostomy in relation to antegrade JJ stenting involve discomfort for the patient, a higher risk of infection, and displacement of the external drain^(6-8)^.

Antegrade JJ stenting is a feasible and minimally invasive alternative technique. However, there are few studies in the literature that provide a technical description of antegrade JJ stenting. Therefore, the objective of the current work was to perform a retrospective analysis of the data related to the use of a modified technique and the results of the antegrade JJ stenting procedure in a population of cancer patients at a university hospital. The conventional technique has some limitations in cases in which there is pronounced ureteral neoplastic involvement, which hinders the insertion of the double J catheter over the guidewire. In view of that difficulty, we perceived the need to use the modified technique proposed in this article.

## MATERIALS AND METHODS

This study was approved by the local committee for ethics in research, educational management, and research. Because this was a retrospective study that merely analyzed the database, the requirement for written informed consent was waived.

### Patient selection

The data were obtained from electronic medical records, diagnostic imaging examinations, and laboratory examinations of patients submitted to antegrade JJ stenting for malignant ureteral obstructions in the department of interventional radiology of the institution, between March 1, 2017 and May 31, 2019. Patients submitted to antegrade JJ stenting for benign causes were excluded from the study. Patients were assigned numbers to ensure the confidentiality of information and to protect their privacy.

The antegrade JJ stenting procedures were performed by an interventional radiologist with seven years of experience and a resident in radiology and diagnostic imaging during the second and third years of residency. The absolute contraindications for performing antegrade JJ stenting were uncorrected coagulopathy, absence of a safe pathway, impaired cardiopulmonary function, hemodynamic instability, pregnancy, multiple renal cysts, and severe uncontrolled hypertension.

### Modified antegrade JJ stenting technique

**Figure 1:**
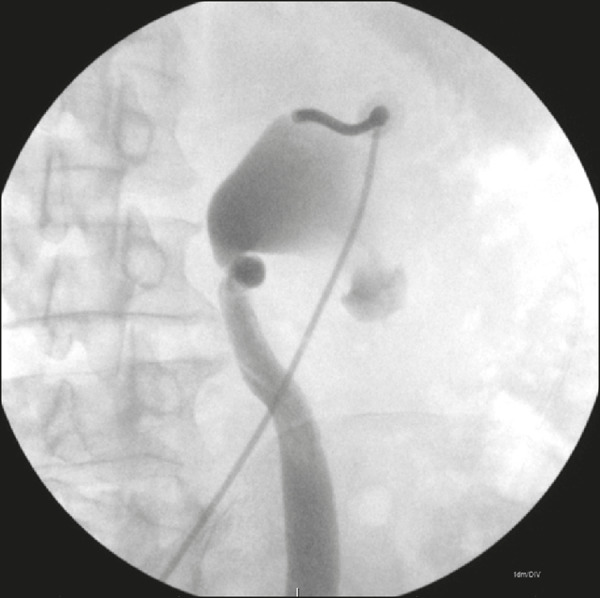
Puncture through the posterior calyx of the upper collecting system of the right kidney, with the patient in left anterior oblique position.

The antegrade JJ stenting procedures were performed either under local anesthesia or under local anesthesia with sedation. After the skin, subcutaneous cellular tissue, and the renal capsule had been infiltrated with 2% lidocaine (10 mL), the percutaneous access to the collecting system was performed with the patient in the left anterior oblique position, regardless of the laterality of the collecting system to be approached. We used an 18-gauge × 15 cm Chiba needle under fluoroscopic and ultrasound guidance, which afforded proper visualization of the insertion, from the skin to the renal calyx, following the Seldinger technique. The location of the renal puncture is dictated by the indication of access, considering the anatomical restrictions^(4,8,9)^. The punctures were normally performed using a posterolateral oblique approach of the upper collecting system, along the avascular plane of Brödel, through the safest and easiest access to the ureteropelvic junction (Figure 1).

**Figure 2:**
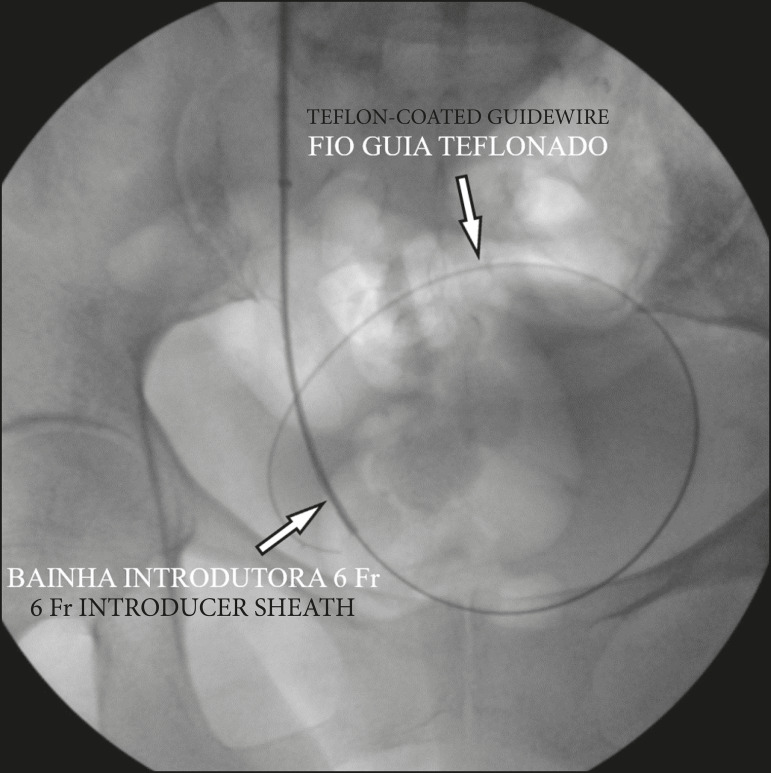
J-tipped Teflon-coated guidewire positioned within the bladder and insertion of the 6 Fr × 45 cm introducer sheath.

After positioning the needle in the renal calyx identified by ultrasound, a urine sample was collected and sent for urine culture. Antegrade pyelography then began with an injection of nonionic iodinated contrast (350 mg I/mL) for a fluoroscopic visualization of the anatomy of the collecting system, with immediate decompression after proper positioning of the needle. A 6 Fr introducer was placed at the ureteropelvic junction using the Seldinger technique. Using a hydrophilic 0.035” guidewire system, we successfully fed a 5 Fr diagnostic catheter past the obstructed point and positioned it within the bladder. We removed the hydrophilic guidewire and positioned a 0.035” J-tipped Teflon-coated guidewire inside the bladder. We then removed the 5 Fr catheter and replaced it with a 6 Fr × 45 cm introducer sheath (Figure 2).

**Figure 3:**
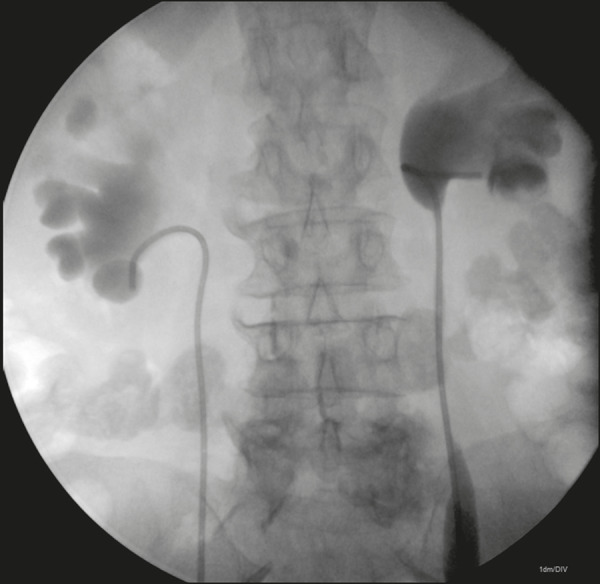
Double J catheters properly positioned in the renal pelves.

**Figure 4:**
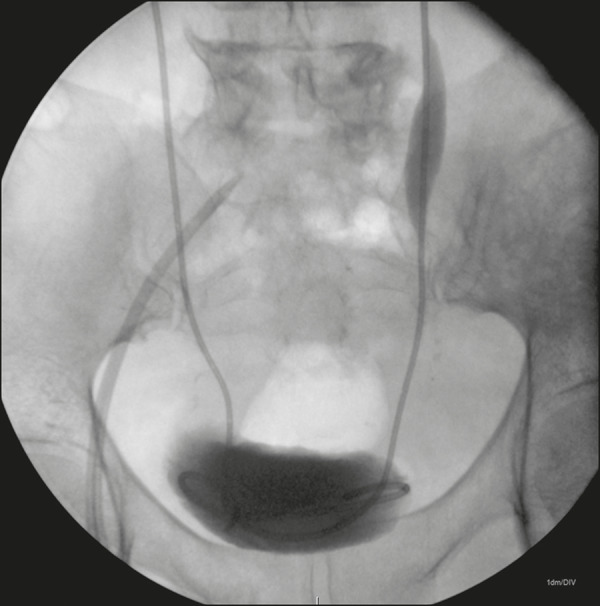
Double J catheters properly positioned inside the bladder.

We fed the double J catheter through the introducer sheath, with or without the Teflon-coated guidewire, and then, with the aid of the sheath dilator, advanced it until the distal end of the double J stent entered the bladder, using fluoroscopic guidance for proper anchoring of the pigtail. We then pulled the introducer sheath back over the dilator until the sheath remained only in the renal pelvis. At that point, with the help of the dilator, we advanced the proximal (renal) end of the double J catheter into the proper position within the collecting system. The properly positioned double J catheters are shown in Figures 3 and 4.

After the antegrade JJ stenting, ultrasound was performed to exclude possible complications. At 24 h after stenting, we took an abdominal X-ray to confirm the positioning of the catheter and the excretion of the contrast agent employed. The urology team replaced the double J catheters every three months under cystoscopic guidance.

### Technical and clinical success

The technical success of the procedure was defined as the maintenance of patency of the urinary pathways with a reduction in the degree of hydronephrosis, as evidenced on imaging examinations (ultrasound or computed tomography). Clinical success was defined as pain reduction and reduced levels of nitrogenous waste, as monitored during hospitalization and outpatient care.

### Complications

Complications were stratified based on the results and were classified as major or minor as defined by the International Society of Radiology^(10)^. Major complications result in hospitalization for treatment (for procedures that had previously been performed on an outpatient basis), an unplanned increase in the level of care, prolonged hospital stay (> 48 h), permanent adverse sequelae, or death. Minor complications do not result in sequelae-they require either no treatment or additional treatment with overnight hospitalization for observation^(10)^.

### Statistical analysis

The data were entered into a 2010 Microsoft Excel spreadsheet. All statistical analyses were performed with the IBM SPSS Statistics software package, version 20.0 (IBM Corp., Armonk, NY, USA).

## RESULTS

During the study period, antegrade JJ stenting was performed in 32 patients: 20 women (mean age, 66.2 years) and 12 men (mean age, 61.5 years). Those patients underwent a total of 53 antegrade JJ stenting procedures, of which 42 were bilateral, seven were in the left collecting system, and four were in the right collecting system. When we evaluated the degree of hydronephrosis, we found it to be severe in 43 cases, moderate in nine, and mild in one.

Of the 32 patients, 22 had post-renal acute kidney injury and ten did not. Fifty procedures were successful and three were not. In two cases, failure was due to migration of the double J catheter, leading to poor urinary drainage by 24 h after the procedure, whereas it was due to an inability to advance the catheter past the obstruction in one case. In the latter case, the extent of the involvement of the middle and distal ureter by the cervical tumor was greater than 10 cm. In the two cases of catheter migration, we opted to remove them via the urethra, using a snare catheter, and then performed percutaneous nephrostomy.

Complications occurred in three patients (one case of lumbar pain, one case of subcapsular hematoma, and one case of pyelonephritis), all of whom had undergone insertion of bilateral ureteral catheters. The patient who developed pyelonephritis presented a favorable evolution after starting a course of parenteral antibiotic therapy. In all 29 of the patients in whom the procedure was successful, there was a reduction in the level of pain, a reduction in the quantity of nitrogenous waste, and an increase in creatinine clearance, whereas there was no improvement in the three patients in whom the procedure failed.

Our technical success rate was 94.3%, the minor complications rate was below 5.6%, and there were no major complications. The data relating to the causes of malignant urological obstructions, to approach, to technical success, to failures, and to complications are summarized in Table 1.

**Table 1:** Etiology of malignant urological obstructions, together with the distribution of approaches, technical success, failures, and complications.

Indication	Patients	Unilateral approach	Bilateral approach	Technical/clinical success	Technical failure	Complications
Cervical cancer	8 (25%)	1	7	7	1	1
Bladder cancer	6 (18.8%)	1	5	6	-	1
Colorectal cancer	5 (15.6%)	2	3	5	-	-
Prostate cancer	5 (15.6%)	-	5	4	1	-
Ovarian cancer	4 (12.5%)	3	1	3	1	1
Retroperitoneal cancers	3 (9.4%)	3	-	3	-	-
Metastatic cancers	1 (3.1%)	1	-	1	-	-
Total	32 (100%)	11	21	29	3	3

A 6 Fr × 26 cm double J catheter was used in 50 procedures, and a 4 Fr × 14 cm double J catheter was used in three procedures. The procedure time ranged from 14 min to 55 min. Among the 32 patients in the study, discharge from the interventional radiology department occurred < 12 h after the procedure in 20, 12-24 h after in nine, and > 24 h after in three. All of the patients were monitored as outpatients for the first 60 days after discharge.

All of the patients in our sample had been referred to the interventional radiology department from the urology or oncology departments. Of the 32 patients studied, 25 had been submitted to an attempted retrograde insertion of a double J catheter by the urology team, without success. In the seven remaining patients, there had been no such attempt; those patients opted directly for antegrade JJ stenting.

## DISCUSSION

There is no consensus in the literature about the management of ureteral obstruction due to malignancies, in which the choice of technique should be individualized according to the clinical condition of the patient, the degree of urgency, the materials available, the experience of the interventional radiologist, and the wishes of the patient^(1)^. Drainage by retrograde insertion of a double J catheter under cystoscopic guidance has technical limitations associated with the location and extent of the tumor, with high rates of failure when the obstruction is secondary to pelvic or retroperitoneal tumors-cases in which the percutaneous approach provides better results^(1,10)^.

In our study, the main oncological cause of ureteral obstructions was invasive cervical cancer, followed by bladder cancer. Romero et al.^(11)^ obtained similar results. In the study conducted by Venyo et al.^(6)^, the main obstructive neoplasm of the ureter was bladder cancer, followed by prostate cancer.

Punctures performed using a posterolateral oblique approach to the upper collecting system, along the renal avascular plane (Brödel’s line), allow easier access to the ureteropelvic junction and facilitates catheter manipulation in the direction of the ureter, as well as providing a safe, relatively avascular, puncture route^(4,8,12)^. In antegrade pyelography with iodinated contrast injection and fluoroscopic visualization of the anatomy of the collecting system, decompression should be performed immediately after the proper positioning of the needle because, especially in patients with infected urine, because allowing the collecting system to be overly distended could lead to bacteremia^(6)^. In the present study, we observed that in punctures made through the upper calyces, positioning the rigid J-tipped guidewire and the 6 Fr × 45 cm introducer sheath inside the bladder resulted in straightening of a tortuous ureter in cases of megaureter, making it easier to insert the double J catheter. After the insertion of the catheter, the proximal pigtail of the ureteral catheter may not be formed at the beginning; however, it generally forms within a few days.

In our experience, the technical difficulty in cases of malignant ureteral obstruction is not in feeding the hydrophilic guidewire through the tortuosities of the upper and middle ureter, which makes it necessary to use a catheter with a specific curvature to overcome these tortuosities, as proposed by Lee^(13)^, because we concluded that the technical difficulty in such cases could be resolved by altering the location of the renal puncture site. Punctures performed in the medium and lower calyces required a sharp angle from the puncture site in relation to the renal pelvis and upper ureter, hampering the passage of the double J catheter even with a rigid guidewire. That is why we always recommend that access be through the upper calyces, which provide better angulation, thus avoiding that problem.

The technical difficulty to be overcome, which motivated the use of the modified technique proposed in this article, is related to advancing the double J catheter through a point of obstruction resulting from neoplastic involvement, as well as to the appropriate positioning in the bladder. We therefore passed a 6 Fr × 45 cm sheath, on a rigid guidewire, beyond the obstructive point and into the bladder, allowing the double J catheter to be inserted from inside the sheath, with or without the use of a guidewire and without suffering external resistance caused by ureteral narrowing.

When urgent relief of the obstruction is the single determining factor, percutaneous nephrostomy seems to be the most reliable approach in the scenario of malignancies, with lower chances of loss of drainage patency in the long term. However, the disadvantage of percutaneous nephrostomy is the need to use a collecting pouch, which has to be replaced regularly, causing discomfort and decreasing the quality of life of the patient, in addition to the increased risk of infection and displacement of the external drain^(6,10)^, which often results in the patient initially refusing the procedure.

Major complications associated with antegrade JJ stenting reportedly occur in 4-8% of cases and include the following^(6,14)^: heavy bleeding, which can be treated with angiographic embolization; inadvertent puncture of the pleura or abdominal viscera (bowel loops, liver, or spleen); and septicemia. Inadvertent damage to the intestine is a rare complication, occurring when the colon is in a retrorenal position^(1)^. Pleural complications, including pneumothorax, hemothorax, empyema, and hydrothorax, occur in less than 0.2% of the patients^(1)^. Minor complications, including retroperitoneal extravasation of urine, capsular hematoma, and macroscopic hematuria, reportedly occur in 3-15% of cases ^(7)^. Mild hematuria, resulting from urothelial irritation, is common after placement of a ureteral catheter. Significant hematuria after placement of a ureteral catheter can be caused by an ureteroarterial fistula between the ureter and the common or internal iliac artery. This rare phenomenon was reported in the context of pelvic cancer treated with surgery and radiation^(1)^. Knowledge of the anatomy and vascularization of the kidneys is of vital importance to select a safe route for percutaneous puncture and to reduce the risk of complications^(6,8-10)^. According to Ganatra et al.^(7)^, the main complications are pain, macroscopic hematuria, and infection/urosepsis. Minor complications, specifically lumbar pain, self-limited subcapsular hematoma, and pyelonephritis, occurred in three (5.6%) of the patients evaluated in the present study.

A malignant extrinsic ureteral obstruction is a potentially complex clinical problem for urologists and oncologists, who often collaborate in the care of these patients^(15)^. In our clinical experience, the two medical specialties differ not only in their recommendations for treatment but also in their concerns about complications. In cases of failure of antegrade JJ stenting for unilateral obstruction, oncologists preferred percutaneous nephrostomy as the next option, whereas urologists preferred manipulation of the double J catheter-replacement, repositioning, or placement of a second catheter^(15)^. Urologists and oncologists agreed that ureteral catheters are more comfortable and provide a better quality of life than does percutaneous nephrostomy. Urologists reported that the greatest risk of percutaneous nephrostomy is displacement, while oncologists reported that infection was the greatest risk. Specialists in both fields were prone to recommend changing ureteral catheters every three months. There was no difference between the two specialties regarding overall satisfaction with the current techniques and interest in future techniques that would be less invasive^(15)^.

## CONCLUSION

Antegrade JJ stenting in patients with ureteral obstruction due to malignancy is safe and effective. The technique described in our study is easy to reproduce, can be performed by a trained professional, and does not require the use of general anesthesia. It is well tolerated by adult patients in all age groups and of both genders, with complication rates similar to those of other methods.
